# Lipotoxicity as a therapeutic target in obesity and diabetic cardiomyopathy

**DOI:** 10.3389/jpps.2024.12568

**Published:** 2024-04-19

**Authors:** Michinari Nakamura

**Affiliations:** Department of Cell Biology and Molecular Medicine, Rutgers New Jersey Medical School, Rutgers Biomedical and Health Sciences, Newark, United States

**Keywords:** heart failure, lipotoxicity, obesity, diabetes, diabetic cardiomyopathy, lipotoxic cardiomyopathy, cardiometabolic disease, inflammation

## Abstract

Unhealthy sources of fats, ultra-processed foods with added sugars, and a sedentary lifestyle make humans more susceptible to developing overweight and obesity. While lipids constitute an integral component of the organism, excessive and abnormal lipid accumulation that exceeds the storage capacity of lipid droplets disrupts the intracellular composition of fatty acids and results in the release of deleterious lipid species, thereby giving rise to a pathological state termed lipotoxicity. This condition induces endoplasmic reticulum stress, mitochondrial dysfunction, inflammatory responses, and cell death. Recent advances in omics technologies and analytical methodologies and clinical research have provided novel insights into the mechanisms of lipotoxicity, including gut dysbiosis, epigenetic and epitranscriptomic modifications, dysfunction of lipid droplets, post-translational modifications, and altered membrane lipid composition. In this review, we discuss the recent knowledge on the mechanisms underlying the development of lipotoxicity and lipotoxic cardiometabolic disease in obesity, with a particular focus on lipotoxic and diabetic cardiomyopathy.

## Introduction

Lipid metabolism plays a pivotal role in diverse physiological processes in the heart [1]. Obesity, defined as an increased body mass index (BMI) resulting from abnormal or excessive fat accumulation, is a disease that impairs health. Obesity perturbs lipid metabolism across nearly all tissues, giving rise to ectopic and excessive lipid accumulation, where lipids become toxic, termed lipotoxicity. Overweight and obesity are commonly linked to cardiac diseases, including cardiac hypertrophy, remodeling, and cardiomyopathy [2, 3]. By focusing on the intricate pathways that govern lipid metabolism, there is potential for therapeutic interventions to improve cardiovascular outcomes in patients with cardiovascular disease (CVD) [4–6].

**FIGURE 1:**
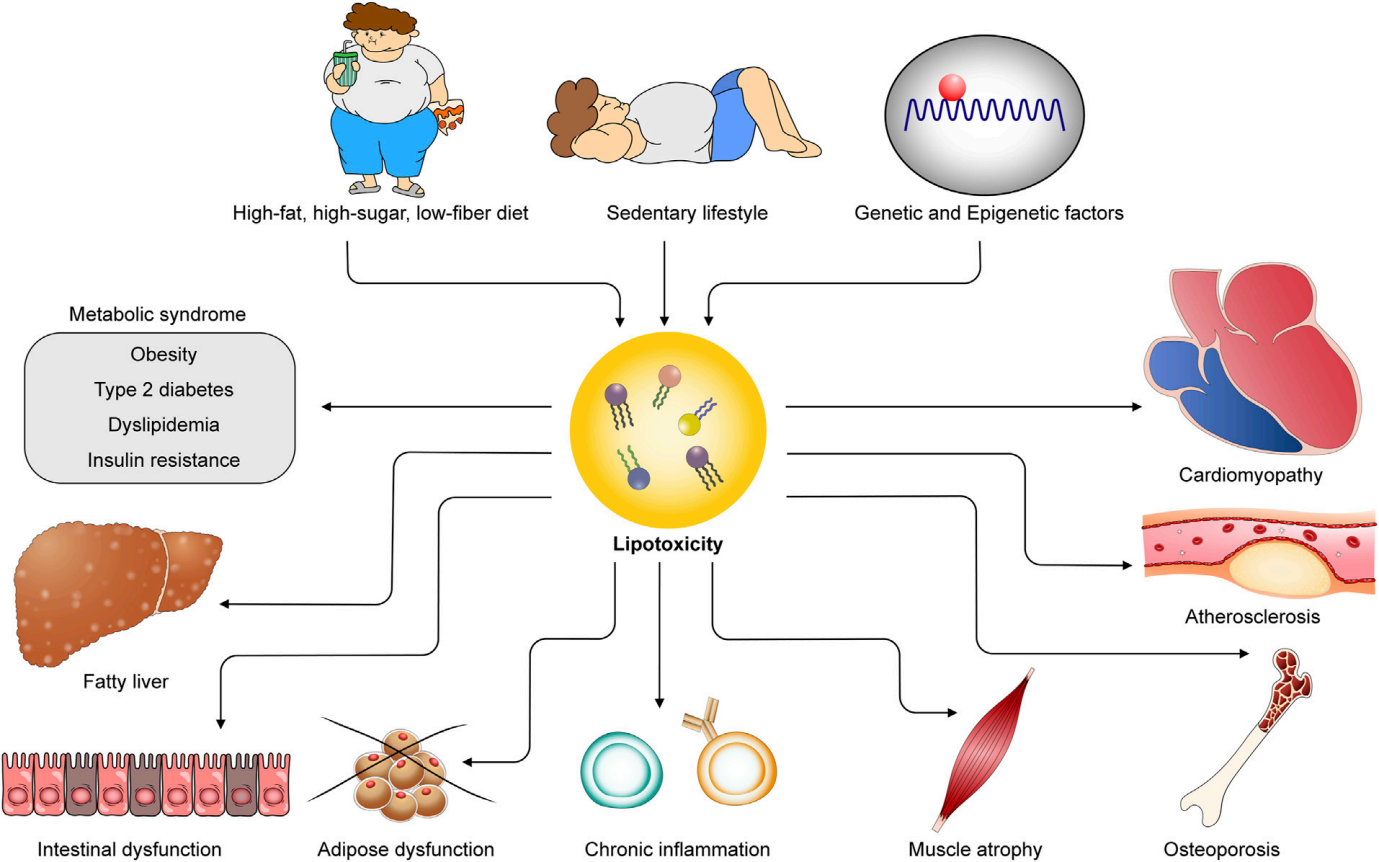
Obesogenic environments and genetic factors contribute to lipotoxicity, leading to cardiometabolic diseases. Unhealthy diets, sedentary lifestyles, and genetic and epigenetic influences provoke excess lipid accumulation in both adipose and non-adipose tissues, giving rise to lipotoxicity. Lipotoxicity underlies the pathogenesis of diverse cardiometabolic diseases, including metabolic syndrome, cardiomyopathy, atherosclerosis, fatty liver disease, intestinal dysfunction, adipose dysfunction, chronic inflammation, and osteoporosis.

The population with overweight or obesity is increasing worldwide [7]. Based on the NCD Risk Factor Collaboration, 2 billion adults (39% of the world’s adult population) were estimated to be overweight and 671 million (12% of the world’s adult population) of whom had obesity. In the United States, the prevalence of being either overweight or obese is 36.8% in children and adolescents and the age-adjusted prevalence of overweight or obesity is 71.2% in adults [8]. The clinical diagnosis of obesity is made with a BMI ≥30 kg/m^2^ or ≥27.5 kg/m^2^ for people of Asian origin, calculated as weight in kilograms divided by height in meters squared. Individuals who are overweight or obesity are more prone to developing hypertension, dyslipidemia, type 2 diabetes, fatty liver disease, osteoarthritis, cancers, obstructive sleep apnea, and CVD (Figure 1). Every 5 kg/m^2^ increase in BMI is associated with 41% increased risk for the development of heart failure [8]. However, according to the fact sheets provided by the World Health Organization, obesity is not merely a risk factor, but rather classified as a disease^1^. Obesity is linked to higher mortality rate [9]. All-cause mortality is lowest at about 22.5–25 kg/m^2^ of BMI and higher degrees of obesity are associated with progressively premature mortality with a reduced median survival by 8–10 years at class 3 obesity, defined as a BMI 40 to <45 kg/m^2^, due mainly to CVD [10]. The data presented strongly support the notion that targeting obesity should be a priority in order to prevent cardiometabolic disease.

**FIGURE 2:**
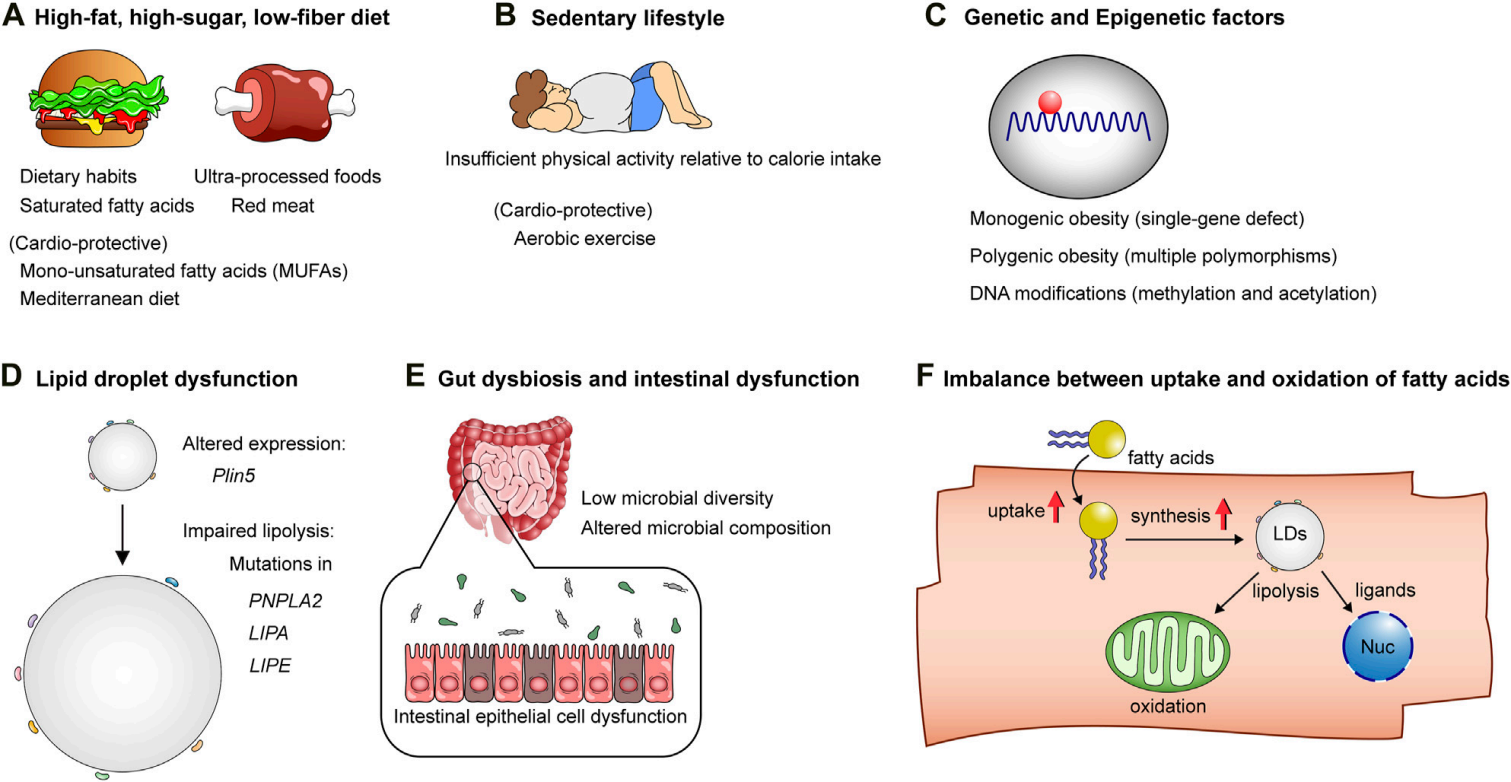
The mechanisms underlying obesity and lipotoxicity. **(A)** High-fat, high-sugar, low-fiber diets: Unhealthy dietary habits and patterns, characterized by saturated fatty acids, ultra-processed foods, and red meat, contribute to the onset of obesity and cellular lipotoxicity, while diets rich in mono-unsaturated fatty acids (MUFAs) and Mediterranean diets are considered healthy alternatives, counteracting the lipotoxic effects of unhealthy dietary patterns. **(B)** Sedentary lifestyle: Insufficient physical activity relative to calorie intake predisposes individuals to obesity. **(C)** Genetic factors and DNA modifications: These factors influence susceptibility to obesity and lipotoxicity-related cardiometabolic diseases. **(D)** The biology of lipid droplets: Lipid droplets play a pivotal role in maintaining the homeostasis of lipid metabolism. Mutations or altered expression of genes related to lipid droplet formation or lipolysis can result in lipotoxicity. **(E)** Intestinal epithelial cell dysfunction, low diversity of gut microbiota, and altered microbial composition affect host lipid metabolism, thereby contributing to obesity and lipotoxic tissue injuries. **(F)** Increased fatty acid uptake and lipid droplet synthesis without adequate facilitation of fatty acid oxidation in mitochondria, as well as ligand-dependent and -independent transcriptional activation in the nucleus, result in excessive lipid accumulation in cells, leading to lipotoxicity.

The pathogenesis of obesity is complex and multifactorial (Figure 2). The traditional studies that search genetic variants for obesity susceptibility with a combination of genetically altered mouse models (“The human obesity gene map”) unveiled the relationship between single-gene mutations and obesity, as well as elucidated certain causal links between them [11] (Figure 2C). Genome-wide association studies over the past two decades have identified a number of genetic loci associated with obesity and estimated that common genetic variants may account for >20% of the variation in BMI [12]. Monogenic obesity, which arises from chromosomal deletions or single-gene defects, is typically a rare condition that follows a Mendelian pattern of inheritance. It is characterized by early-onset and severe obesity. Conversely, polygenic obesity, also referred to as common obesity, is attributed to the influence of multiple polymorphisms. Each genetic locus is considered to have a small effect on the susceptibility to obesity. However, recent studies on gene discovery have uncovered a striking similarity in the underlying biological mechanisms between monogenic and polygenic obesity (reviewed in Ref. [13]). These studies have unequivocally demonstrated the critical role of genetics in contributing the variation in BMI. Future investigation is necessary to elucidate how specific genetic loci and their interactions impact biological processes, ultimately leading to an increased susceptibility to obesity.

In the general population, environmental factors, collectively known as the “obesogenic environment,” are the primary contributors to overweight and obesity (Figures 2A, B). These factors encompass a range of elements, including dietary habits, levels of physical activity, income, and education. More specifically, the composition of diets, dietary patterns, fat compositions in diets, and the calorie balance between calorie intake and expenditure all play significant roles in determining an individual’s weight status. Fat is an essential component of dietary nutrients, alongside protein, carbohydrate, vitamin, and mineral. Fat provides the highest calories per Gram, with 9 calories per Gram, compared to carbohydrate and protein which provide 4 calories per Gram. Dietary fats consist of saturated, unsaturated, and polyunsaturated fatty acids. Saturated fatty acids are generally regarded as unhealthy fats, while polyunsaturated fatty acids are considered to be healthier fats. A double-blind, parallel-group, randomized trial (LIPOGAIN) showed that 7-week overconsumption of muffins high in saturated fatty acids leads to significant increases in hepatic and visceral fat storage compared with overconsumption of those high in n-6 polyunsaturated fatty acids (PUFAs), despite both groups experiencing similar weight gain in healthy individuals [14]. Randomized controlled trials and meta-analysis suggest that replacing dietary saturated fats with unsaturated fats, especially polyunsaturated vegetable oils, reduces serum cholesterol by 15–20% and CVD by ∼30% [15–17]. Further, while there is no universally accepted definition for ultra-processed foods [18], population-based prospective cohort studies and meta-analyses have consistently shown an association between the consumption of ultra-processed foods and the development of diabetes [19], obesity, and CVD [20] and all-cause mortality [21–23]. Collectively, these studies provide compelling evidence that both environmental and genomic factors contribute to the susceptibility of humans to overweight and obesity, which disrupt the homeostasis of lipid metabolism, consequently giving rise to deleterious effects on cardiometabolic health.

In this review, we will discuss the intricate interactions between lipotoxicity and cardiomyopathy in the context of obesity. This review will provide a comprehensive overview of the current understanding of factors associated with obesity, the impact of obesity on lipid metabolism, the mechanisms underlying abnormal and excessive lipid accumulation in cells that induces cellular toxicity and cardiomyopathy, as well as potential therapeutic approaches targeting lipotoxicity for CVD.

## Lipid metabolism in health

Lipids are a diverse group of organic compounds, including fats, oils, hormones, waxes, and cell membrane components. Fats are a specific type of lipids primarily composed of triesters formed from fatty acids and glycerol. Dietary fats supply humans with essential source of fatty acids, which act as cellular membrane components, molecules of energy storage, substrates of adenosine triphosphate (ATP) production in mitochondria, and signaling molecules. Dietary fats, predominantly composed of triglycerides, undergo digestion by lipase to generate fatty acids and monoglycerides in the intestinal lumen. These components are then absorbed by enterocytes, where they are re-esterification into triglycerides and packaged into chylomicrons. Chylomicrons are subsequently secreted into the lymphatic system and transported through the large vessels.

Circulating fatty acids are taken up by various organs, including skeletal muscle, heart, and liver. When nutrients are abundant, fats are primarily stored in adipocytes within adipose tissue for long-term storage, while the liver serves as short-term storage. During fasting, adipose tissue releases fatty acids to be used by peripheral tissues. Additionally, brown and beige adipose tissues, specialized forms of adipose tissue, utilize lipids to generate heat and maintain body temperature. Adipocytes can store large amounts of lipids in the form of lipid droplets, preventing abnormal lipid accumulation in other tissues. However, when lipids exceed the storage capacity of lipid droplets within adipocytes, such as in obesity and diabetes, excessive lipids lead to ectopic intracellular lipid deposition in other tissues. Consequently, adipose tissue dysfunction is closely linked to obesity and other cardiometabolic disorders, including diabetes, insulin resistance, and CVD. In the heart, an oxidative tissue, lipids are stored similarly in the form of lipid droplets as a means to sequester toxic lipids. This mechanism serves as a form of cardioprotection, as demonstrated by experiments conducted on rodents. The transgenic mice that overexpress diacylglycerol acyltransferase 1 (DGAT1), an enzyme that catalyzes the conversion of diglycerides and fatty acyl-CoA to triglycerides, in cardiomyocytes using α-myocyte heavy chain promoter doubled the myocardial triglyceride content but reduced the levels of ceramides and diglycerides, which are considered as toxic lipids, with normal contractile function [24]. Overexpression of long-chain acyl-CoA synthetase (ACS) in cardiomyocytes leads to lipotoxic cardiomyopathy in mice, a phenotype similar to diabetic cardiomyopathy [25]. However, the detrimental effect was rescued when these mice were crossed with DGAT1 transgenic mice [24]. These findings suggest that triglycerides may not be inherently toxic, and lipid droplets formation actually serves as a protective mechanism for the heart. On the other hand, cardiomyocytes generate ATP by oxidizing long-chain fatty acids (LCFAs) in mitochondria, with a significant portion of these LCFAs being derived from lipid droplets [26]. This highlights the crucial role of lipid droplets biology in maintaining the balance of lipid metabolism and overall homeostasis.

## Lipid droplets as a physiological lipid storage organelle

In a state of nutrient surplus, lipids are stored in intracellular organelles, called lipid droplets, which are composed of a hydrophobic core of neutral lipids, mainly triglycerides and cholesteryl esters, encircled by a phospholipid monolayer with integral and peripheral proteins [27]. Lipid droplets are primarily formed in adipocytes of adipose tissues during fed conditions. However, they can also be found in essentially every cell type of other tissues to protect the cells from lipid-induced toxicity by buffering excessive amounts of toxic lipids (Figure 2D). Depending on the status of cellular metabolism, lipid droplets are assembled on the endoplasmic reticulum (ER) through a series of processes. Lipid droplets are generated; 1) from neutral lipids, most commonly triglycerides made by DGAT1/2 and sterol esters made by acyl-CoA: cholesterol *O*-acyltransferases (ACAT1/2); 2) lipid droplet budding facilitated by ER membrane phospholipid composition and some proteins, such as fat storage-inducing transmembrane proteins (FIT1/2), Pln1 (mammalian perilipins), and Seipin; 3) growth and maturation of lipid droplets by droplet-droplet fusion or transfer of triglyceride to lipid droplets; and 4) targeting of integral and peripheral proteins to lipid droplets. High-confident proximity labelling approach has identified around 150 proteins on the lipid droplet monolayer [27–29]. Among them, the perilipin (PLIN) protein family has been extensively studied as a key regulator of hydrolysis to facilitate the release of fatty acids for ATP production.

Lipid droplets dynamically, structurally, and functionally interact with other cellular organelles, including mitochondria, peroxisomes, and lysosomes, playing a crucial role in facilitating diverse functions of lipid droplets [27, 30]. The interaction with mitochondria could provide sites for trafficking of fatty acids hydrolyzed from lipid droplets to mitochondria for ATP production via β-oxidation in response to starvation in mouse embryonic fibroblasts (MEFs) [31]. This interaction also provides protection to mitochondria in MEFs by promoting triglyceride synthesis through DGAT1 and reducing fatty acid incorporation into other toxic lipid species, including acylcarnitines, preventing their exposure to mitochondria [32]. A recent study demonstrated that efficient lipid droplets-to-mitochondria fatty acid trafficking and β-oxidation require Ser155 phosphorylation of PLIN5 and its direct interaction with the mitochondrial fatty acid transport protein 4 (FATP4) during myoblast starvation [33]. The interaction between lipid droplets and mitochondria has been observed to decrease in failing human hearts compared to donor hearts, as assessed by transmission electron microscopy [34], which may potentially contribute to the reduced fatty acid utilization in mitochondria in failing hearts. In brown adipocytes, as opposed to other cell types, peri-lipid droplet mitochondria increase pyruvate oxidation and ATP synthesis capacity with reduced β-oxidation capacity, which contributes to promoting triglyceride synthesis and lipid droplet expansion [35]. Lipid droplets also interact with peroxisomes, membrane-enclosed organelles that carry out oxidation reactions, including fatty acids β-oxidation [30]. A recent study [36] demonstrated that the lipid droplets and peroxisome network mediates the longevity effect of dietary mono-unsaturated fatty acids (MUFAs), rich in the Mediterranean diet that is linked with increased human lifespan and decreased CVD [37, 38]. Papsdorf et al. used *Caenorhabditis elegans* with a combination of genetics and lipidomics analyses and found that MUFAs upregulate the numbers of lipid droplets and peroxisomes with decreased lipid oxidation in the intestinal cells [36]. This contributes to decreasing lipid membrane damage and preserving membrane integrity during ageing, thereby driving lifespan extension. Additionally, lipid droplets interact with lysosomes via perilipin 2/3 (PLIN2/3) and chaperone-mediated autophagy machinery [30], which promotes lipid droplet and neutral lipid turnover with elevated levels of adipose triglyceride lipase (ATGL) and autophagy proteins during starvation [39]. These findings strongly indicate that the biology of lipid droplets plays a central and pivotal role in maintaining lipid metabolism.

It has been reported that lipid droplets can also be formed directly from the inner nuclear membrane, serving as a site for lipid storage in high-fat conditions in yeast cells [40]. This process is regulated by Seipin through detection of phosphatidic acid and diglyceride enrichment at the inner nuclear membrane [40]. Nuclear lipid droplets may also contribute to nuclear envelope expansion and regulation of gene expression by sequestering transcription factors on lipid droplets, including Opi, an ER-associated transcription factor, which may affect cellular lipid metabolism [40]. In contrast, another study demonstrated that nuclear lipid droplets in hepatocytes are derived from lipoprotein precursors present in the ER membrane in a Seipin-independent manner [41], indicating species and cell-type specific mechanisms of nuclear lipid droplets formation. Nuclear lipid droplets appear functionally distinct from cytoplasmic lipid droplets, although their precise role remains largely unknown.

Depending on cellular metabolism, demands, and nutrient availability, such as nutrient deprivation, esterified lipids stored in lipid droplets within adipose tissue undergo hydrolysis via lipolysis, a catabolic process of lipid droplets, or lipophagy, a specific form of autophagy that selectively degrades cytoplasmic lipid droplets, to liberate fatty acids and sterols into the bloodstream [42]. Lipolysis is stimulated by the binding of sympathetic nervous system-mediated catecholamine to β-adrenergic G-protein-coupled receptors [43]. Fatty acids released from lipid droplets serve as a crucial resource for diverse cellular processes in peripheral tissues, including ATP production, membrane biogenesis during periods of high demand for membranes, and acting as mediators of signaling pathways [27]. Lipolysis is regulated by three major enzymes; ATGL [also known as Patatin-like phospholipase domain-containing protein 2 (PNPLA2)], a rate-limiting enzyme that catalyzes the hydrolysis of triglycerides to diglycerides; abhydrolase domain containing 5 (ABHD5, also known as CGI-58), an essential coactivator of ATGL; and hormone-sensitive lipase (HSL), an enzyme that mediates hydrolysis of diglycerides. Monoacylglycerol lipase (MGL) hydrolyzes monoglycerides to generate glycerol and fatty acids. Lipophagy, also known as acid lipolysis, is another form of intracellular pathway responsible for the triglycerides degradation that takes place in lysosomes. Lysosomal acid lipase (LAL) is an enzyme essential for the hydrolysis of triglycerides and cholesteryl esters within lysosomes. LAL deficiency is an autosomal recessive disease caused by mutations in the *LIPA* gene. Wolman’s disease is a severe disorder characterized by dyslipidemia, severe hepatosteatosis, hepatosplenomegaly, and premature death during infancy. Cholesteryl ester storage disease (CESD) is a less severe disorder that manifests dyslipidemia, atherosclerosis, and coronary artery disease due to enhanced foam-cell formation [44]. GWAS identified single nucleotide polymorphisms in the *LIPA* gene that associate with coronary artery disease [45, 46].

Compared to the knowledge of lipid droplets in adipose tissue and liver, the pathophysiological role of lipolysis in the heart is limited [47]. LCFAs released from lipid droplets act as the primary fuel source for oxidative ATP generation, ligands for nuclear hormone receptors, and substrates for synthesis of membrane lipids in cardiomyocytes. Mutations in either *PNPLA2* or *ABHD5* gene in humans cause neutral lipid storage disease with myopathy (NLSDM), characterized by systemic accumulation of triglycerides in lipid droplets [48], which often requires heart transplantation due to severe cardiomyopathy. Homozygous frameshift mutations in the *LIPE* gene encoding HSL impair triglyceride catabolism, although the clinical manifestations are less pronounced than that of NLSDM. Patients with defective HSL display dyslipidemia, systemic insulin resistance, diabetes, and hepatic steatosis (partial lipodystrophy) [49]. Failing hearts, primarily due to non-ischemic cardiomyopathy, exhibit a 0.64-fold reduction in mRNA expression of *Plin 5*, the encoded protein preferentially expressed in highly oxidative tissues such as the heart, compared to donor hearts [34]. Systemic deletion of *Plin 5* results in the absence of myocardial lipid droplets and diminishes myocardial triglyceride levels, exacerbating age-related cardiomyopathy due to increased oxidative stress, a condition mitigated by antioxidant therapy with *N*-acetylcysteine (NAC) [50]. Conversely, overexpression of PLIN5 in cardiomyocytes via the α-MHC promoter results in enlarged and increased lipid droplets with massive triglyceride accumulation (3.5-fold increase) in the heart. This occurs through direct inhibition of ATGL-mediated lipolysis [51, 52], leading to concentric hypertrophy with preserved systolic function in 4-month-old male mice fed a normal chow diet [53]. PLIN5 cardiac-specific transgenic (cTg) mice show exacerbated cardiac hypertrophy and reduced systolic function when fed a HFD [54]. However, intriguingly, PLIN5 cTg mice display resistance to developing obesity and glucose intolerance under a HFD, presumably due to enhanced β-adrenergic signaling in adipose tissue, compared to WT mice. The role of MGL in the heart has not been thoroughly investigated. Pharmacological blockade of MGL with JZL184 increases systemic levels of 2-arachidonoylglycerol (2-AG), a class of signaling lipids called endocannabinoids. This increase enhances the myocardial recruitment of neutrophils and monocytes through the upregulation of neutrophil recruiting chemokines, CXCL1 and CXCL2, after myocardial infarction, thereby exacerbating inflammation, infarct size, and cardiac dysfunction in mice [55]. These results suggest the role of MGL in the myeloid cell recruitment from the bone marrow to the heart and subsequent cardiac inflammation via regulating endocannabinoid catabolism. These findings highlight the pathophysiological importance of effectively regulating lipid droplets in lipid metabolism and overall cardiometabolic health.

## The impact of gut microbiota on host lipid metabolism and obesity

The gastrointestinal (GI) tract is the digestive system, which facilitates the movement of food and liquids through peristalsis. It also plays a crucial role in the digestion and absorption of catabolized nutrients in the intestine, such as amino acids derived from proteins, simple sugars (glucose, fructose, and sucrose) derived from carbohydrates, and fatty acids and glycerol derived from fats. In addition to enzymatic digestion, the GI tract harbors a diverse community of microorganisms, called gut microbiota, which not only helps digestion of food but also modulates the effects of dietary nutrients on host physiology and disease [56, 57] (Figure 2E). Genomic analysis of fecal samples from obese and lean twins demonstrated the significant association between the diversity of gut microbiota and obese and lean phenotypes in humans [58]. Transplantation of fecal microbiota from obese humans increases body mass and adiposity in mice fed a low-fat high-fiber diet than those from lean humans [59]. Importantly, transplantation of fecal microbiota from lean humans increase fermentation of short-chain fatty acids and decrease branched-chain amino acids metabolism compared to those from obese humans in mice. The low diversity of gut microbiota is associated with long-term dietary habits characterized by low consumption of fruits, vegetables, and fishery products in obese or overweight subjects [60]. Additionally, in the same study, dietary intervention aimed at reducing calorie intake for weight loss have been shown to improve gut microbial diversity, particularly in individuals with initially low microbial diversity, and clinical phenotypes such as insulin resistance and elevated blood triglycerides and high-sensitivity C-reactive protein levels, especially in individuals with initially high microbial diversity [60]. These clinical studies provide clear evidence of the significant relationship between gut microbiota and the cardiometabolic health of the host human.

**FIGURE 3:**
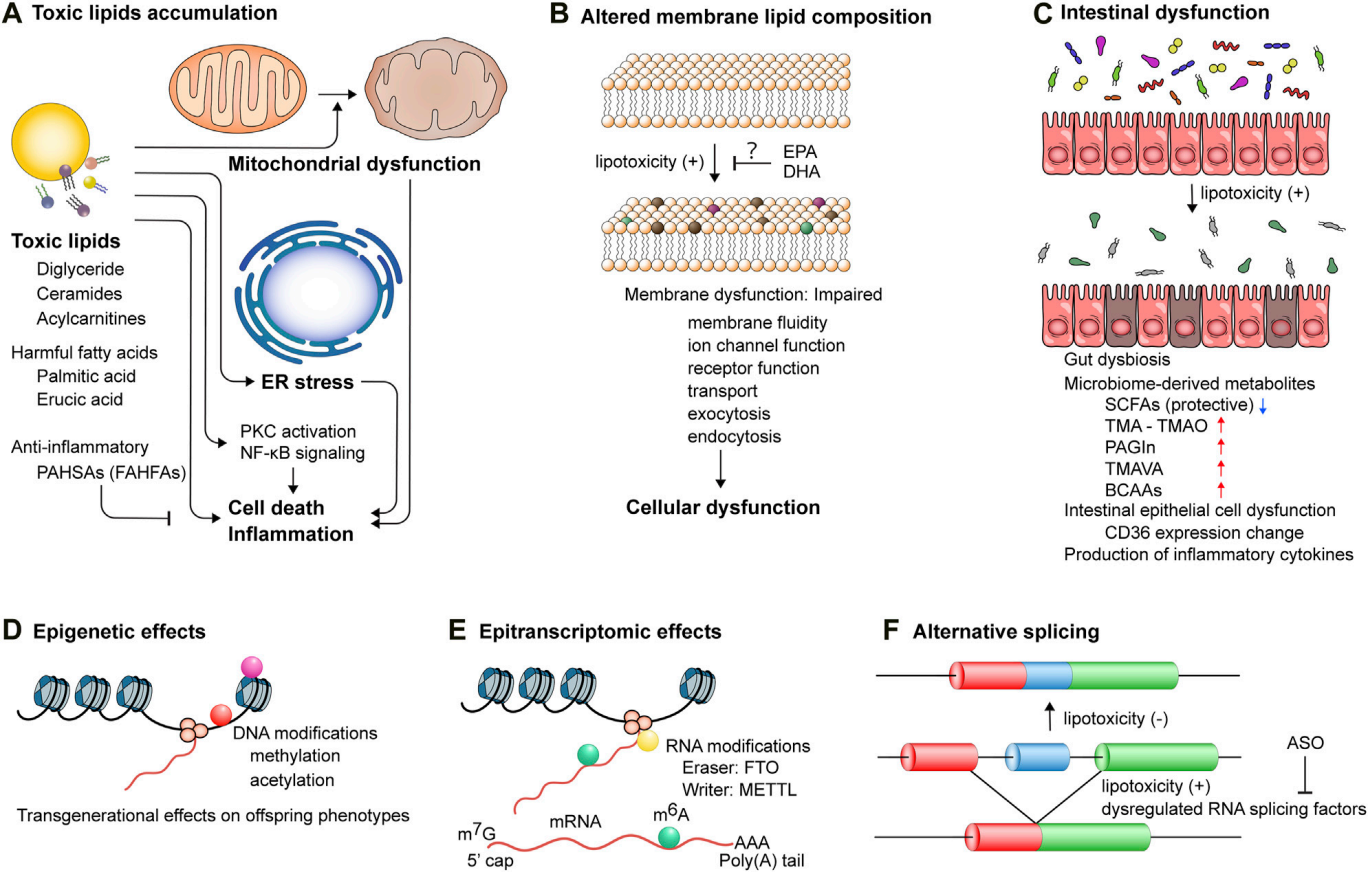
The mechanisms underlying lipotoxicity-induced cellular and tissue dysfunction. **(A)** Accumulation of toxic lipids triggers mitochondrial dysfunction, endoplasmic reticulum (ER) stress, and activation of protein kinase c (PKC) and NF-κB signaling pathways, leading to cell death and inflammation. PAHSAs, palmitic acid esters of hydroxy stearic acids; FAHFAs, branched fatty acid esters of hydroxy fatty acids. **(B)** Lipotoxicity perturbs membrane lipid composition, which impairs membrane functionality, including membrane fluidity, ion channel function, receptor function, transport, exocytosis, and endocytosis, thereby inducing cellular dysfunction. EPA, eicosapentaenoic acid; DHA, docosahexaenoic acid. **(C)** Intestinal epithelial cell dysfunction and gut dysbiosis induce lipotoxicity and also augment the production of microbiome-derived metabolites, such as trimethylamine (TMA), phenylacetylglutamine (PAGIn), NNN-trimethyl-5-aminovaleric acid (TMAVA), and branched chain amino acids (BCAAs), while reducing the production of short-chain fatty acids (SCFAs). **(D)** DNA modifications, including methylation and acetylation, exert transgenerational effects on offspring cardiometabolic phenotypes. Lifestyle interventions of parents can potentially reverse these modifications. **(E)** Epitranscriptomic modifications of post-transcriptional mRNA regulate gene expression. Aberrant deposition of RNA modifications, such as *N* ^6^-methyladenosin (m^6^A), mediated by dysregulated proteins, such as fat mass and obesity associated (FTO) and methyltransferase-like (METTL), is implicated in the pathogenesis of cardiometabolic diseases. **(F)** Toxic lipid overload induces alternative splicing events, which can be modulated by antisense oligonucleotides (ASOs) targeting pre-mRNA splicing.

Dietary fats impact gut microbiota population, which in turn plays fundamental roles in host lipid metabolism (Figure 2E). Various diseases develop by microbial dysbiosis, where the gut microbial communities are imbalanced or lack diversity without regard to the presence or absence of harmful or beneficial microbes [61, 62]. A recent study demonstrated the effects of different fatty acid compositions in dietary fats on gut microbiota composition in humans. Schoeler et al. showed that individuals consuming lower amounts of saturated fatty acids exhibit higher microbial diversity compared to those consuming higher amounts of saturated fatty acids, with no significant association observed between the amount of MUFAs or PUFAs consumption and microbial diversity [63]. In addition, the study showed a negative correlation between gut microbial diversity and the degree of liver steatosis, as determined by magnetic resonance imaging, in both obese and lean individuals, alongside a positive correlation between dietary intake of saturated fatty acids and MUFA and the fatty liver index, only in obese individulas [63]. It is known that individuals with metabolic syndrome, including obesity and type 2 diabetes, exhibit altered gut microbial features [64]. Two large-scale studies have identified unique gut microbiome and serum metabolome features as risk factors to develop ischemic heart disease in humans. These features include decreases in gut microbial density and serum levels of short-chain fatty acids (SCFAs) as well as an increase in the production of branched-chain amino acids [65, 66], the changes commonly observed in individuals with obesity and diabetes [64]. On the contrary, the Mediterranean diet, characterized by a low intake of animal-derived foods and a high intake of plant-based fatty acids, was associated with a higher capacity of host gut microbiota to produce SCFAs and a lower risk of cardiometabolic disease in humans [67]. These data suggest that SCFAs produced by the gut microbiota may play a significant role in the interplay between gut microbiota and cardiometabolic health [68] (Figure 3).

In addition to SCFAs, certain gut-derived metabolites have been demonstrated to correlate with host cardiometabolic health (Figure 3C). Gut microbiota-derived trimethylamine (TMA) is absorbed in the intestine and oxidized in the host liver to form trimethylamine *N*-oxide (TMAO). The impact of elevated circulating TMAO levels on the development of CVD, particularly atherosclerosis, has been identified in humans [69] as well as its effects on platelet hyperactivation in human platelets, incident risk for thrombotic events in humans and a carotid artery injury mouse model, and mortality in human subjects [70, 71]. A recent study using cryopreserved human heart specimens revealed a substantial accumulation of TMAO in the myocardium of ischemic cardiomyopathy (fold change = 2.2) compared to donor myocardium [72]. It is noteworthy that this distinction attained statistical significance only in the male myocardium of ischemic cardiomyopathy (fold change = 1.95) and dilated cardiomyopathy (fold change = 1.96) compared to male donor hearts. Conversely, the female myocardium displayed no significant difference between cardiomyopathy and donor hearts [72], indicating a sex-specific metabolic regulatory mechanism in the gut-heart axis. Dietary choline and L-carnitine, abundant in red meat and containing a trimethylamine structure, alter gut microbial composition, significantly increasing TMA synthesis and circulating TMAO levels in mice [73]. A recent study showed that a HFD impairs mitochondrial bioenergetics in the host colonic epithelium, leading to increased luminal bioavailability of oxygen and nitrate. This alteration in turn amplifies respiration-dependent choline catabolism by E. *Coli*, thereby enhancing host circulating TMAO levels [74]. This study emphasizes the significance of colonocyte dysfunction induced by a HFD. Furthermore, through untargeted metabolomics, phenylacetylglutamine (PAGIn) has been identified as a gut microbiota-derived metabolite that activates platelets through G-protein coupled receptors, including α2A, α2B, and β2-adrenergic receptors, with its circulating levels positively associated with thrombosis risk, CVD, and major adverse cardiovascular events in humans [75]. In another study, untargeted metabolomics analysis using prospective heart failure cohort samples revealed that N,N,N-trimethyl-5-aminovaleric acid (TMAVA), derived from trimethyllysine through the gut microbiota, is significantly elevated in hypertensive individuals, with its plasma levels positively associated with incident cardiac death [76]. TMAVA treatment exacerbated 12-week HFD-induced cardiac hypertrophy and dysfunction in mice by inhibiting fatty acid oxidation through the reduction of carnitine metabolism and subsequently increasing myocardial lipid accumulation [76]. These findings indicate that the Western dietary habit, characterized by excess ingestion of red meat and a high-fat, low-fiber diet, increases the risk of CVD, including atherosclerosis and lipotoxic cardiomyopathy, in part through alterations of gut microbial compositions and gut microbiota-derived metabolites.

Moreover, a multi-omics study demonstrated that increased fecal carbohydrate metabolism, particularly monosaccharides, in the gut microbiome contributes to the development of insulin resistance, accompanied by an increase in inflammatory cytokines [77]. This is consistent with a previous finding that excessive monosaccharides promote ectopic lipid accumulation and low-grade inflammation [78–80]. The metabolic reprogramming of immune cells, their dysregulation, and the concomitant changes in cytokine production in the intestinal epithelium in response to a HFD feeding play crucial roles in the development of chronic low-grade inflammation in obesity and diabetes (reviewed in Ref. [81]). Using T cell-specific Myd88 knockout mice, which exhibit defective T follicular helper cells, it has been demonstrated that T cell-dependent immunity protects mice from diet-induced obesity by reducing lipid absorption through a secreted microbial molecule-mediated regulation of host *CD36* gene expression [82]. Another recent study revealed that dietary sugar induces gut dysbiosis, eliminating T helper (Th) 17 cells. The Th17 cells regulate lipid absorption across the intestinal epithelium by suppressing *CD36* expression in an IL-17-dependent manner. Consequently, the elimination of intestinal Th17 cells exacerbates the development of metabolic syndrome induced by a high-fat, high-sugar diet in mice [83]. Furthermore, integrated multi-omics analyses have revealed the associations between the usage of drugs, including statins and metformin, and diversity of the host gut microbiota in humans [84]. The data suggests that certain kinds of medication may have the potential to influence cardiometabolic disease by modulating the population of gut microbiota. Additionally, there is an association between host genetic factors and microbial diversity. However, it is crucial to note that environmental factors, particularly dietary habits, have a more significant impact on gut microbial diversity [85]. In summary, these studies provide compelling evidence that diets high in saturated fatty acids and sugar, and low in fiber, lead to a reduction in gut microbial diversity and density, which in turn produce more harmful and fewer beneficial fatty acid metabolites and alter immune cell population and function. Consequently, these alterations contribute to the development of cardiometabolic disease through impaired lipid metabolism and inflammation.

## Molecular mechanisms by which excessive lipid accumulation leads to lipotoxicity

Lipids constitute essential components of normal cellular biology; however, their excessive accumulation within cells can give rise to toxicity. Accumulated toxic lipids induce ER stress [86], oxidative stress, and mitochondrial dysfunction, accompanied by impaired autophagy and mitophagy [87], thereby causing cell death and inflammation [2]. It is noteworthy that the impact of free fatty acids on cellular functions varies, with not all being uniformly detrimental or beneficial at comparable levels. Circulating free fatty acids are elevated in obesity and diabetes, concomitant with increased myocardial fatty acid uptake and decreased glucose uptake. Imaging tools, such as positron emission tomography and magnetic resonance spectroscopy, enable the visualization and quantification of changes in human myocardial substrate and energy metabolism associated with metabolic syndrome (reviewed in Ref. [88]). Here we discuss the recent studies that provide insight into the molecular mechanisms as to how the storage of lipids is facilitated beyond physiological levels and how lipids become toxic in cells and tissues.

### Imbalance between fatty acid uptake/storage and utilization

When nutrients are abundant, such as during feeding and in metabolic syndrome, cardiomyocytes increase the uptake of fatty acids, storing them for later use. Prolonged high-fat diet (HFD) consumption leads to the development of cardiac hypertrophy and contractile dysfunction in mice, a phenotype similar to diabetic cardiomyopathy [89]. A recent study demonstrated that palmitic acid, but not oleic acid, activates GSK-3α, a serine/threonine protein kinase, in the nucleus in cardiomyocytes, which phosphorylates peroxisome proliferator-activated receptor α (PPARα) at Ser280, located in the ligand binding domain [90]. This phosphorylation reaction stimulates fatty acid uptake and storage, but not β-oxidation, leading to an imbalance between lipid uptake and utilization in cardiomyocytes. Initially, this signaling manifests as physiological to facilitate the storage of energy source; however, due to the limited capacity for lipid droplet storage in the heart, persistent activation of this pathway, as observed in prolonged exposure to a HFD, induces myocardial ectopic lipid accumulation, resulting in diabetic cardiomyopathy in mice [90]. These findings suggest that 1) GSK-3α senses the availability of fatty acids, particularly saturated fatty acids, stimulating lipid uptake and storage through biased activation of PPARα, and that 2) the lipid storage signaling depends on the saturation level of fatty acids [90] (Figure 2F). In line with this finding, increasing fatty acid oxidation by acetyl coenzyme A carboxylase 2 (ACC2) deletion specifically in cardiomyocytes prevents HFD-induced diabetic cardiomyopathy in mice, indicating that increasing fatty acid oxidation alone does not impair cardiac function. This beneficial effect is, in part, mediated by the upregulation of parkin-mediated mitophagy, contributing to the restoration of mitochondrial function [91]. These findings underscore the significance of maintaining a balance between lipid uptake and utilization in the heart, serving as a preventive measure against diabetic cardiomyopathy.

### Toxic lipid accumulation

Intramyocardial lipids that exceed the capacity for storage and utilization can be toxic (Figure 3A). It is important to note that the pathogenesis of diabetic cardiomyopathy is multifactorial, including toxic lipid accumulation and toxic glucose metabolites. For example, metabolic intermediates of glycolytic pathways (e.g., dihydroxyacetone phosphate, methylglyoxal, and glucose-6 phosphate) either generate advanced glycation end products (AGEs) or facilitate the biosynthesis of glycoproteins (protein O-GlcNAcylation), which leads to the excessive accumulation of NADPH [2, 92, 93]. Among these multifactorial components, cardiac steatosis stands out as a clinical hallmark of diabetic cardiomyopathy and serves as an independent predictor of diastolic dysfunction [94, 95]. Importantly, cardiac steatosis precedes the onset of diabetes and the manifestation of systolic dysfunction [96]. Several lines of transgenic mice, especially those overexpressing long-chain acyl-CoA synthetase in cardiomyocytes [25], fatty acid transport protein 1 [97], lipoprotein lipase (LpL) [98], or PPARα [99] using the α-myosin heavy chain promoter, exhibited similar phenotypes to diabetic cardiomyopathy. These include excessive myocardial lipid accumulation, cardiac hypertrophy, and contractile dysfunction, leading to premature death. These rodent studies provide convincing evidence that increased ectopic lipid accumulation in the heart is sufficient to induce lipotoxic or diabetic cardiomyopathy independently of systemic metabolism. A recent study using hyperpolarized ^31^P pyruvate tracer with ^1^H and ^13^C in noninvasive magnetic resonance spectroscopy analysis has confirmed increased myocardial lipid accumulation and impaired pyruvate metabolism and energetics in the heart of diabetic patients with diastolic dysfunction [100]. It is noteworthy that the size and number of lipid droplets may be a surrogate marker of lipotoxicity since neither lipid droplet nor triglyceride *per se* appears harmful in cells. It is considered that lipid droplets serve as a mechanism for sequestrating toxic lipids away from other organelles, including mitochondria and ER. The primary questions center on precisely defining the identities of toxic lipids and elucidating their roles in cellular function. Lipid metabolism intermediates, such as diglycerides, ceramides, and acylcarnitines, have been documented as toxic lipids and extensively reviewed elsewhere [2, 101] (Figure 3A).

Briefly, diglyceride is a lipid metabolite that acts as a second messenger to activate protein kinase C (PKC) by binding to the C1B domain. PKC phosphorylates a plethora of cellular substrates, including insulin receptor substrate 1, calcium channel, titin, and nuclear factor-κB (NF-κB) subunits, which leads to insulin resistance, inflammation, oxidative stress, Ca^2+^ overload, and cell death, thereby giving rise to the development of cardiac hypertrophy and dysfunction [102]. Ceramides are a family of lipid composed of sphingosine covalently linked to a fatty acid. Ceramide production is increased by various kinds of cellular stresses by three major pathways: *de novo* synthesis, sphingomyelin hydrolysis, and the salvage pathway [103]. Ceramides induce insulin resistance by inhibiting Akt signaling in mice [104] and activate the mitochondrial apoptotic pathway via increased membrane permeability [105, 106]. Lipotoxicity in LpL transgenic mouse heart was ameliorated by pharmacological inhibition of *de novo* ceramide biosynthesis with myriocin or crossing with genetic haploinsufficiency of LCB1 mice, a subunit of serine palmitoyltransferase (SPT). This was evidenced by the amelioration of cardiac dysfunction and the prolonged survival rate, implying that ceramide accumulation can be cardiotoxic, thereby contributing to the pathogenesis of lipotoxic cardiomyopathy [107]. Intraperitoneal injection of a SPT I inhibitor, myriocin, reduced intramyocardial levels of ceramides as well as diglycerides in mice, which was associated with improved cardiac glycolysis rates, as assessed by an isolated working heart perfusion model, in the setting of diet-induced obesity [108]. Acylcarnitines are intermediate oxidative metabolites composed of a fatty acid esterified to a carnitine molecule, synthesized by the enzymes carnitine palmitoyltransferase 1 and carnitine palmitoyltransferase 2. Acylcarnitines serve as carriers to transport long-chain fatty acids (LCFAs) across the mitochondrial membrane. Accumulation of acylcarnitine through the inhibition of DGAT1 induces mitochondrial uncoupling and dysfunction, indicating the protective role of DGAT1-mediated lipid droplet biosynthesis, possibly by acting as a buffer against lipotoxic acylcarnitines [32]. A metabolic profiling of blood samples obtained simultaneously via invasive catheterization from the aortic root and coronary sinus in patients with severe aortic stenosis and hypertrophic cardiomyopathy revealed myocardial accumulation of long-chain acylcarnitines, presumably due to suppressed fatty acid oxidation, in the heart with aortic stenosis [109]. Additionally, circulating levels of long-chain acylcarnitines were independently associated with measures of maladaptive left ventricular remodeling in patients with severe aortic stenosis [110].

The amounts of free fatty acids, the degree of fatty acid saturation, and the cellular fatty acid compositions also represent critical factors in determining toxicity. In a recent unbiased analysis encompassing 61 structurally diverse fatty acids, the integration of transcriptomics, lipidomics, cell morphological features, and functional profiling has identified a subset of 20 fatty acids that could be toxic to β-cells [111]. Among these fatty acids, the well-known toxic fatty acid, palmitic acid, was included, serving as a substrate for the *de novo* synthesis of ceramides. Consistent with prior reports, oleic acid was identified as a protective fatty acid. Among 20 toxic fatty acids, 12 fatty acids were MUFAs, including erucic acid [111]. It is important to note that MUFAs are generally regarded as non-toxic or healthy fatty acids. Erucic acid was found to associate with decreased membrane fluidity, ER stress, and cell death. The data presented indicates that relying solely on traditional criteria based on saturations categories (saturated, monounsaturated, and polyunsaturated fatty acids) does not adequately define the lipotoxic potential of fatty acids. Piccolis et al. conducted transcriptomic, lipidomics, and proteomics analyses with genome-wide short hairpin RNA (shRNA) screening and have demonstrated that palmitate, a saturated fatty acid, increases saturated glycerolipids and ER stress in human leukemia cells, where di-saturated glycerolipids play a central role in lipotoxicity [112]. Furthermore, genome-wide shRNA screen identified more than 350 genes as genetic modifiers of lipotoxicity. Among these genes, the deletion of ER-localized glycerol-3-phosphate acyltransferase (GPAT) or the putative E3 ubiquitin ligase RNF213 was found to be sufficient to protect cells from lipotoxicity [112], indicating that these proteins are critical downstream targets of lipotoxicity.

It is noteworthy that fatty acids act not only as membrane components and energy substrates but also possess anti-inflammatory and anti-diabetic properties. Recently, branched fatty acid esters of hydroxy fatty acids (FAHFAs) have been identified as endogenous lipids in mammals [113], exhibiting unique biological functions [114]. Various FAHFAs display distinct biological activities, with some demonstrating anti-inflammatory and anti-diabetic effects in both murine models and humans [113, 115] (reviewed in Ref. [116]). Levels of palmitic acid esters of hydroxy stearic acids (PAHSAs), a subfamily of FAHFAs, are reduced in the serum and adipose tissue of individuals with insulin resistance [113, 117]. Long-term administration of PAHSAs improves insulin sensitivity and glucose tolerance in mice, partly by activating the G protein-coupled receptor 40 (GPR40), a long-chain fatty acid receptor [117]. Furthermore, PAHSAs decrease β-cell inflammation in mice by attenuating ER stress through a glucagon-like peptide 1 receptor (GLP-1R) and mitogen-activated protein kinase signaling [118]. A recent study has identified ATGL as a biosynthetic enzyme for FAHFAs by esterifying an FHA with a fatty acid from triglyceride or diglyceride [119], underscoring the significance of transacylase activity in ATGL alongside its lipase function. Considering the detrimental effects of functional knockdown of ATGL on cardiac morphology and function, the potential beneficial effects arising from ATGL-mediated release of FAHFAs may hold significance in understanding cardiac physiology. Given the anti-inflammatory properties of FAHFAs, there emerges a critical need to elucidate the functional roles of both endogenously produced and exogenously administered FAHFAs in cardiac pathologies in the context of obesity.

### Altered membrane lipid composition

Lipids constitute the main component of cellular membranes, thereby regulating biological processes through modulation of membrane properties (such as membrane fluidity, ion channel and receptor function, and transport), as well as influencing the processes of exocytosis and endocytosis of molecules [120]. Altered membrane lipid composition and function result in cellular dysfunction (Figure 3B). For example, docosahexaenoic acid (DHA), a PUFA, in glycerophospholipids (GPLs) reduce membrane bending rigidity, contributing to functional endocytosis. In contrast, lipids with long and saturated fatty acids (e.g., sphingolipids) induce thicker and less fluid membranes [121, 122]. The distinct effects of eicosapentaenoic acid (EPA) and DHA treatment on atherosclerosis, membrane oxidation, membrane lipid dynamics and fluidity, as well as downstream lipid metabolite function, have been reviewed in Ref. [123]. A recent study unveiled that increased lipid saturation and exogenous saturated fatty acid overload rigidify the nuclear envelope and ER membranes, thereby fostering nuclear envelope rupture [124]. This paper showed that lipid acyl chain unsaturation with balanced lipid saturation is required for nuclear pore complex integrity and nucleocytoplasmic transport. In this regard, lipid droplets can buffer saturated membrane lipids to preserve nuclear envelope architecture. Collectively, these findings indicate that an increase in fatty acid saturation leads to changes in membrane lipid composition, triggering ER stress and the pathway of cell death.

### Epigenetic effects of lipotoxicity

Maternal and paternal obesity, prolonged exposure to unhealthy diets, and diabetes predispose offspring to develop metabolic syndrome even in a healthy lifestyle [125] through epigenetic modifications (Figure 3D). These include S-adenosylmethionine-mediated methylation and Acetyl-CoA or NAD^+^-dependent (de)acetylation, leading to transgenerational effects on offspring phenotypes [126–129]. Huypens et al. have conducted *in vitro* fertilization of embryos using sperm and oocytes from parental (F0) mice fed a HFD into healthy foster mothers to generate offspring (F1), followed by a HFD challenge [130]. This study demonstrated that female, but not male, F1 offspring from obese parents exhibit more pronounced increases in body weight and fat mass compared to those from lean parents. This indicates the presence of epigenetic germline inheritance of diet-induced obesity and insulin resistance. In addition, Wan et al. demonstrated that HFD-induced deregulation of lipid metabolism and lipid accumulation are transmitted to multigenerational progeny in *C. elegans* through nuclear receptors NHR-49 (a functional homolog of mammalian peroxisome proliferator-activated receptor α) and NHR-80 (a homolog of mammalian hepatocyte nuclear factor 4) and transcription factors SBP-1 and DAF-16, which are conferred by histone H3K4 trimethylation [131]. Importantly, DNA methylation of 4,875 Cytosine-phosphate-guanine (CpG) sites was differently affected between a 7-week excessive intake of saturated fatty acids and PUFAs in human adipose tissue. This underscores the distinct impacts of dietary fatty acid composition on epigenetic changes [132]. These findings provide evidence of transgenerational inheritance of obesity and diabetes even preceding pregnancy. Moreover, a growing body of evidence indicates that the nutritional status of parents may predispose their offspring to diabetic cardiomyopathy. In *Drosophila*, HFD-induced diabetic cardiomyopathy was transmitted to two subsequent generations, a phenomenon associated with increased systemic H3K27 trimethylation and the downregulation of ATGL and PGC-1 [133], a regulatory network modulating lipotoxicity in the heart [134]. These findings provide compelling evidence of intergenerational inheritance of cardiometabolic disease, including diabetic cardiomyopathy.

Epigenetic modifications can be reversible through lifestyle interventions. In a recent study, the genome-wide DNA methylation analysis was conducted using cord blood DNA collected from the Treatment of Obese Pregnant women (TOP)-study populations. This study has demonstrated that lifestyle intervention during pregnancy in women with obesity has an obvious impact on DNA methylation, 379 sites in 370 genes, in cord blood, which is notably linked to the body composition in the offspring [135]. The genes containing lifestyle intervention-related DNA methylation significantly associate with gene ontology biological processes for striated muscle cell proliferation, response to fatty acid, and adipose tissue development. In another study, Son et al. showed that maternal exercise enhances brown adipogenesis and thermogenesis through increased DNA demethylation in the *Prdm16* promoter via an exercise-induced apelin/α-KG-dependent axis in mice. This modification mitigated offspring obesity when challenged with a HFD [136]. These findings underscore the critical impact of obesity and lifestyle interventions on the development and prevention of cardiometabolic disease, not only for oneself but also future generations.

### Epitranscriptomic effects of lipotoxicity

In addition to epigenetic modifications of histone and DNA, epitranscriptomic modifications of post-transcriptional mRNA play a pivotal role in gene expression by regulating RNA stability, subcellular localization (such as cytoplasmic export of RNAs containing methyladenosine), and alternative splicing (such as the efficient recognition of splice sites by methyladenosine) [137] (Figure 3E). Internal *N* ^6^-methyladenosine (m^6^A) modifications and the 5′ cap are the most prevalent modifications in mRNA. Aberrant deposition of RNA modifications has been implicated in the pathogenesis of human diseases, including neurological deficits, cancer, obesity, and diabetes, by, for example, causing RNA degradation or structural changes (reviewed in Ref. [138]). Fat mass and obesity associated (FTO) is one of the well-studied epitranscriptomic regulators. A genome-wide association study initially identified single nucleotide polymorphisms in the first and second introns of the *FTO* gene as being associated with increased BMI and a predisposition to obesity that persists from childhood into old age, thereby increasing the susceptibility to diabetes [139, 140]. The FTO expression is elevated in mice fed a HFD [141]. Genetic deletion of *FTO* in mice leads to postnatal growth retardation and a significant reduction in adipose tissue by enhancing energy expenditure and systemic sympathetic activation [142], while ubiquitous overexpression of FTO results in obesity by increasing food intake [143]. FTO has been reported as a demethylase of N6, 2′-O-dimethyladenosine (m^6^A_m_) in the 5′ cap, which controls mRNA stability [144], and internal m^6^A_m_ modifications. The m^6^A-sequencing in human diabetic islets revealed several hypomethylated transcripts, including insulin secretion pathway [145]. Furthermore, reduced m^6^A levels by Mettl14 β-cell specific deletion in mice recapitulated the islet phenotype in human diabetes. Additionally, endothelial cell-specific deletion of FTO attenuated retinal vascular endothelial dysfunction and inflammation against streptozotocin-induced diabetes in mice [146]. The methylated RNA immunoprecipitation sequencing (MeRIP-Seq) analysis combined with RNA-Seq has elucidated that FTO represses the *TNFAIP3 interacting protein 1* (*Tnip1*) mRNA expression by erasing m^6^A methylation in the 3′-UTR of *Tnip1* mRNA, thereby increasing NF-κB activity.

Aberrant RNA modifications have been detected in the cardiac tissues of patients with CVD, including atherosclerosis, heart failure, cardiac hypertrophy, and cardiomyopathy. The m^6^A methyltransferase Mettl3 and m^6^A RNA methylation levels are increased in endothelial cells in both *in vitro* and *in vivo* atherogenic environments, resulting in hypermethylation of m^6^A sites predominantly at coding sequences near the 3’ UTR of NLRP1 and KLR4 mRNA [147]. The short-hairpin (sh)RNA-mediated knockdown of Mettl3 effectively prevented atherosclerotic lesion formation in the ApoE^−/−^ mouse model. The levels of m^6^A RNA methylation are increased in both human failing hearts [148] and rat neonatal cardiomyocytes following serum stimulation [149]. The αMHC-promoter-driven overexpression of Mettl3 in cardiomyocytes was sufficient to induce physiological cardiac hypertrophy, while cardiac-specific deletion of Mettl3 exacerbated aging-related and pressure overload-induced cardiac remodeling in mice, concomitant with reduced cardiomyocyte hypertrophy [149]. A recent study demonstrated that cardiac-hypertrophy-associated piRNA (CHAPIR), a PIWI-interacting noncoding RNA (piRNA), directly interacts with Mettl3 to inhibit the m^6^A RNA methylation of *Parp10* mRNA. This methylation enhances PARP10 expression in cardiomyocytes, facilitating pathological cardiac hypertrophy in response to pressure overload [150]. Conversely, while FTO is downregulated in both human and mouse failing hearts, adeno-associated virus (AAV)-mediated upregulation of FTO attenuated contractile dysfunction in response to myocardial infarction through FTO-mediated demethylation and stabilization of the *SERCA2a* mRNA transcript [148]. Mettl14 expression is decreased in both H9C2 cells treated with high glucose and streptozotocin-induced diabetic hearts in rats [151]. Lentivirus-mediated upregulation of Mettl14 attenuated diabetes-associated heart dysfunction by augmenting the m^6^A RNA methylation of long noncoding RNA (lncRNA) TINCR and subsequently suppressing pyroptosis through inhibition of NLRP3 mRNA expression [151]. These results underscore the essential role of m^6^A RNA methylation in maintaining cardiac homeostasis. Collectively, these findings implicate that translational changes related to obesity and diabetes across various cell types are, in part, modulated by mRNA modifications.

### Lipotoxicity-mediated alternative splicing

Alternative splicing provides the complexity of transcriptomes, allowing species, tissue, and cell type-specific regulation of diverse processes. The 2 landmark papers were published in 2012 that address the fundamental question as to what generates differences in organs between species [152, 153]. Although tissue-specific gene expression is highly conserved among vertebrates, these papers demonstrated that alternative splicing patterns are dominated by species-specific differences. The article by Merkin et al. also demonstrated that differential splicing, rather than the abundance of protein kinase, primarily influences the regulation of protein kinase activity by including or excluding exons related to kinase reactions [153]. Global and gene-specific modulations of alternative splicing regulate a wide range of physiological and pathological processes, such as cell death, cell differentiation, and metabolism. These mechanisms contribute to the development of neurological and developmental disorders, CVD, and cancer [154]. The Genotype-Tissue Expression (GTEx) project has identified detailed gene expression regulation with genetic rare variants and alternative splicing for the majority of genes across human tissues by conducting DNA sequencing and multi-tissue RNA sequencing. The GTEx consortium identified functional rare genetic variation and cell type-specific genetic regulation of gene expression [155]. By generating a large number of human full-length well-known proteins and their novel spliced isoforms, Yang et al. demonstrated the protein-protein interaction profiling using yeast two-hybrid screens and a protein complementation assay for validation in human HEK293T cells [156]. This study clearly demonstrated that the majority of isoform pairs share less than 50% of their interactions, and the interaction partners are expressed in a highly tissue-specific manner. These findings indicate that alternatively spliced transcripts may function as distinct proteins rather than minor variants of each other. Nevertheless, the functional implications of genetic rare variations and alternatively spliced variants in the context of human diseases remain largely unknown.

In a recent rodent study, Keller et al. disrupted the 5’ alternative splicing site in the *Bcl2l1* gene to inhibit alternative splicing of *Bcl-x short-isoform* (*Bcl-xS*) in mice *in vivo*, and demonstrated that the suppression of Bcl-xS induces systemic inflammation, splenomegaly, cardiac fibrosis, and cardiomyopathy. This study suggests that *Bcl-xS* alternative splicing is essential for maintaining organ functions in a tissue-specific manner [157]; however, whether this alternative splicing impacts on lipotoxicity and diet-induced cardiomyopathy and, if so, how it regulates lipid metabolism remain to be investigated. Importantly, RNA-sequencing analysis has identified altered expression of 17 RNA splicing factors (e.g., SRSF3) and alternative splicing of 3,525 transcripts corresponding to 2,858 genes in human islet cells in response to palmitate *in vitro* [158] (Figure 3F). The data presented suggests that β-cell dysfunction may be attributable to lipotoxicity-induced alternative splicing. Furthermore, Vernia et al. have identified an alternative splicing program in adipose tissue in response to a HFD challenge in mice *in vivo*. Mice fed a HFD displayed widespread changes in alternative splicing in adipose tissue, a phenomenon correlated with a reduction in the expression of NOVA splicing factors following HFD consumption [159]. Adipocyte-specific NOVA deficient mice showed increased adipose tissue thermogenesis and less weight gain following a HFD, indicating that NOVA enhances an alternative splicing program that suppresses thermogenesis and promotes diet-induced obesity. A recent study using genomic and proteomic analyses has identified enhanced spliceosome proteins expression and alternative splicing machinery in the liver of mice fed a HFD [160]. A major isoform of splicing factor RBFOX2 expression and its activity were suppressed in the liver of diet-induced obese mice, which deregulates alternative splicing of lipid-regulatory genes, impairing cholesterol metabolism [160]. These findings suggest that targeting RNA splicing could be a potential therapeutic approach for mitigating lipotoxicity-induced tissue damage. However, further research is required to elucidate the mechanisms by which excessive lipid accumulation induces alternative splicing events and to determine whether and how alternatively spliced variants in the heart or other tissues contribute to diabetic cardiomyopathy in the context of obesity.

## Clinical relevance of lipotoxicity in cardiovascular disease

Cardiovascular disease in individuals with metabolic syndrome, including obesity, insulin resistance, diabetes, and dyslipidemia, manifests as coronary artery disease (e.g., angina pectoris and myocardial infarction), cardiomyopathy, left ventricular hypertrophy, systolic and/or diastolic dysfunction, arrhythmia, and valvular heart disease [2]. The presence of metabolic syndrome, particularly with an increasing number of comorbidities, associate with a higher risk of age-dependent development of heart failure [161, 162]. A recent pooled analysis of community-based NHLBI cohorts has demonstrated that higher BMI (overall obesity), abdominal obesity, waist circumference, and fat mass are strongly associated with a greater risk of heart failure development among older adults, particularly among those with diabetes [163]. This indicates the pivotal role of diabetes in modifying the association between obesity and heart failure development. Alternatively, clinical studies have demonstrated that obesity can be a therapeutic target for the prevention of heart failure development. For example, the Look AHEAD trial showed that reductions in fat mass and waist circumference, but not lean mass, by an intensive lifestyle intervention, are each significantly associated with a lower risk of heart failure development but not myocardial infarction in adults with diabetes [164]. Although the etiologies of cardiometabolic diseases are multifactorial, obesity-mediated lipotoxicity plays a critical role in developing cardiometabolic disease. This section discusses the clinical relevance of lipotoxicity in cardiometabolic disease (Table 1).

**TABLE 1:** Lipotoxicity-related cardiomyopathy based on the primary pathogenesis of lipid accumulation.

Obesogenic environment or obesity-related cardiomyopathy	
Disease	Underlying diseases
Diabetic cardiomyopathy	Diabetes and insulin resistance
Non-diabetic lipotoxic cardiomyopathy	Metabolic syndrome in the absence of diabetes and insulin resistance
Ischemic or hypertensive cardiomyopathy	Atherosclerotic coronary artery disease, renal disease, and hypertension
Inherited lipotoxic cardiomyopathy caused by mutations in genes for lipid droplets biology	
Disease	Responsible genes
Triglyceride deposit cardiomyovasculopathy (TGCV), Neutral lipid storage disease with myopathy (NLSDM)	*PNPLA2*
Lipodystrophy syndromes	*AGPAT2*
	*Lpin1*
	*CCTα*
	*Seipin*
	*FIT2*
Cholesteryl ester storage disease	*LIPA*

### Lipotoxicity in cardiometabolic disease

Circulating free fatty acids are elevated in obesity, a consequence of their release from enlarged adipose tissue and inadequate utilization in peripheral tissues. This, in turn, induces β-cell dysfunction and insulin resistance, accompanied by ectopic lipid accumulation in peripheral tissues, all contributing to the pathogenesis of type 2 diabetes [165]. Pancreatic islets consist of multiple cell types. To delineate the islet cell type-specific gene expression in healthy individuals and those with type 1 and type 2 diabetes, single-cell RNA sequencing has been employed through the Human Pancreas Analysis Program. Using these datasets, a recent study revealed that the downregulated genes in β-cells are enriched for processes related to mitochondrial function, with minimal reduction of β-cell numbers in type 2 diabetes. This reduction in genes for mitochondrial function contributes to oxidative stress, impaired insulin secretion, and β-cell dysfunction [166]. These findings align with previous reports [167]. In addition to pancreatic β-cell dysfunction, obesity and diabetes contribute to the accumulation of triglycerides in lipid droplets in adipocytes, which induce adipocyte hypertrophy and hyperplasia, resulting in the rapid expansion of adipose tissue. This, in turn, triggers the production of proinflammatory cytokines and further increases circulating free fatty acids [168]. Lipotoxicity in adipose tissue plays a critical role in establishing a state of chronic inflammation in obesity and diabetes. The complex interactions between metabolic and inflammatory pathways in immune cells and metabolic tissues have recently been extensively studied in the field of immunometabolism [169]. These findings indicate that lipotoxicity underlies the pathogenesis of insulin resistance and diabetes in obesity.

### Diabetic cardiomyopathy

Diabetic cardiomyopathy can be clinically diagnosed by the presence of diabetes and cardiomyopathy in the absence of hypertension and coronary artery disease [2, 170]. It is important to exclude primary cardiomyopathy (e.g., hypertrophic cardiomyopathy, dilated cardiomyopathy, tachycardia-induced cardiomyopathy, arrhythmogenic right ventricular cardiomyopathy, left ventricular non-compaction cardiomyopathy, restrictive cardiomyopathy, ion channelopathies, takotsubo cardiomyopathy, and myocarditis) and cardiomyopathies secondarily developed by coronary artery disease, valvular heart disease, and hypertension. Individuals with overweight, obesity, dyslipidemia, or insulin resistance without diabetes, hypertension, and atherosclerotic cardiovascular disease (ASCVD) may also develop a similar cardiomyopathy. Considering the absence of diabetes, these patients may not be diagnosed with diabetic cardiomyopathy in the clinical setting. These similar metabolism-related cardiomyopathies may be referred to as obesity-related cardiomyopathy, insulin resistance-induced cardiomyopathy, lipotoxic cardiomyopathy, or metabolic cardiomyopathy. Although there may be distinct underlying mechanisms, considering the key role of dysregulated metabolism and lipotoxicity in developing these cardiomyopathies and the frequent overlapping of obesity and diabetes, we collectively refer to these cardiomyopathies as diabetic cardiomyopathy in this review article.

The clinical features of diabetic cardiomyopathy are left ventricular hypertrophy and subclinical evidence of diastolic dysfunction in the early stage and systolic dysfunction in the late stage, which promotes heart failure, especially heart failure with preserved ejection fraction (HFpEF). Importantly, there may be a difference in cardiac morphology and function between obese and non-obese HFpEF patients. The echocardiographic and catheter-based functional analyses revealed that obese patients with HFpEF exhibit more pronounced biventricular remodeling and more severe right ventricular dysfunction compared to their non-obese counterparts with HFpEF [171]. A recent report based on the 7 community-based cohorts have demonstrated that approximately 6.2% of diabetic participants without ASCVD develop heart failure in 5 years, contrasting with a rate of 12.7% among diabetic participants with ASCVD [172]. Risk stratification using NT-proBNP and other cardiac biomarkers (e.g., echocardiography and high-sensitivity cardiac troponin T) has identified high-risk participants for heart failure, although a substantial number of participants initially classified as low risk developed heart failure [172]. This underscores the challenge of accurately predicting the development of heart failure in individuals with metabolic syndrome but without a history of ASCVD.

Clinical studies provide compelling evidence that the prominent accumulation of lipids is a hallmark of diabetic cardiomyopathy. Cardiac ^1^H-magnetic resonance spectroscopy (MRS) analysis revealed that diabetic patients, regardless of obesity status, exhibit increased levels of myocardial triglyceride content and impaired energetics in comparison to normal-weight control subjects. Notably, there is no discernible disparity in the level of cardiac steatosis between diabetic patients with and without obesity [173]. The DMCM-AHEAD prospective study showed a significant early and progressive lipid accumulation in transplanted hearts among diabetic recipients when compared to their non-diabetic counterparts. This indicates that the early pathogenesis of human diabetic cardiomyopathy involves significant lipid accumulation in cardiomyocytes [174]. However, further investigation is warranted to elucidate the precise mechanisms by which excessive lipid accumulation contributes to diabetic cardiomyopathy in humans.

### Triglyceride deposit cardiomyovasculopathy (TGCV)

TGCV is congenital heart disease associated with mutations in the *Patatin-like phospholipase domain containing protein 2* (*PNPLA2*) gene encoding adipose triglyceride lipase (ATGL) [175]. This is also known as neutral lipid storage disease with myopathy (NLSDM). The ATGL-mediated lipolysis of cellular triglycerides plays a pivotal role in modulating the PPARs-PGC-1 complex activity. Deficiency of ATGL specifically in cardiac and skeletal muscle results in excessive lipid accumulation and subsequent cardiomyopathy in mice [176]. The clinical features of TGCV include skeletal myopathy and severe cardiomyopathy, characterized by excessive triglyceride accumulation in the myocardium and smooth muscle cells of coronary arteries. This differs from diabetic cardiomyopathy and ASCVD in two key aspects: first, coronary artery disease is evident in TGCV, and second, the predominant deposition in the arteries of TGCV is triglyceride, as opposed to cholesterol in the arteries of ASCVD [177]. Based on recently published diagnostic criteria, TGCV is classified into primary TGCV, which involves a mutation in the *PNPLA2* gene, and idiopathic TGCV, which lacks a mutation in the *PNPLA2* gene. The latter is likely to overlap with other congenital heart diseases that have mutations in genes related to the hydrolysis of triglycerides [178, 179]. Definite primary TGCV demonstrates the presence of the typical Jordan’s anomaly (apparent vacuoles >1 μm in size) in over 90% of polymorphonuclear leucocytes in peripheral blood smears. The diagnostic criteria for TGCV include: 1) defective triglyceride lipolysis, as assessed by a reduced washout rate (<10%) of ^123^I (iodine)-β-methyl-p-iodophenylpentadecanoic acid (BMIPP), a radiolabeled LCFA analogue, in myocardial scintigraphy: and 2) myocardial accumulation of triglycerides, as assessed by magnetic resonance spectroscopy/computed tomography scan or biopsy [179]. According to the Japan TGCV study group, the mean age of male and female patients at TGCV diagnosis was 63.6 years (range: 24–87) and 68.6 years (range: 33–93), respectively. The prevalence of coronary artery disease and heart failure were 74.9% and 71.0%, respectively. The 5-year overall and cardiovascular event-free survival rates after diagnosis were 71.8% (70.9% for males and 74.2% for females) and 54.0%, respectively [180]. Treatment with a medium-chain triglyceride, tricaprin, reduced triglyceride accumulation and improved contractile dysfunction in an *ATGL* knockout mouse model [181]. Additionally, in an investigator-initiated, multicenter, randomized, double-blind trial (Phase IIa) comprising 17 patients with idiopathic TGCV, 8-week oral administration of tricaprin (1.5 g/day) significantly increased the washout rate of BMIPP after baseline adjustments compared to placebo control, but there were no significant changes in the 6-min walk distance [182].

### Atherosclerosis and coronary artery disease

The ASCVD is linked to lipotoxicity in arterial cells, including smooth muscle cells, endothelial cells, and immune cells. Atherosclerosis is developed by accumulation of cholesterol esters in the lipid droplets of macrophages (foam cells), giving rise to atherosclerosis. Atherosclerosis manifests as coronary artery disease, cerebrovascular disease, renal disease, and peripheral artery disease, which frequently leads to ischemic cardiomyopathy, stroke, and hypertensive heart disease. It is noteworthy that coronary artery disease is one of the exclusion criteria for diagnosis of diabetic cardiomyopathy. The thickening and stiffening of the arterial wall, known as arterial stiffness, as assessed by brachial-ankle pulse wave velocity, can be a risk stratification index for prognosis in patients with type 2 diabetes, irrespective of the presence or absence of coronary artery disease [183, 184]. Cholesterol released from oxidized LDL is esterified to cholesteryl esters by the acyl-CoA cholesterol acyltransferase (ACAT1) and stored in lipid droplets in macrophages, which become foamy (foam cells) in atherosclerotic plaques. Genetic inhibition of *ACAT1* exacerbates atherosclerosis in LDL receptor-deficient mice [185]. Consistent with this finding, the ACAT1 inhibitor, pactimibe, exhibited adverse effects on atheroma volume and may contribute to the progression of coronary atherogenesis in humans [186]. In addition, pactimibe treatment was associated with increases in mean carotid intima-media thickness and major cardiovascular events in patients with familial hypercholesterolemia [187]. These data indicate that the formation of cholesteryl esters in the lipid droplets in macrophage represents an adaptive response to hypercholesteremia. It is important to emphasize that vascular smooth muscle cells also accumulate cholesteryl esters, exhibiting features reminiscent of macrophage-derived foam cells in atherosclerotic plaques in the human coronary artery. Smooth muscle cell-derived foam cells account for 50% of the total foam cell population, underscoring the significant contribution to smooth muscle cells to the storage of excess cholesterol [188]. A recent study has demonstrated that the expression of transforming growth factor-β2 (TGF-β2) is more closely linked to the content of smooth muscle cells in human atherosclerotic tissue, showing an inverse association with plaque rupture and inflammation. The data presented indicates the role of smooth muscle cell TGF-β2 in stabilizing plaque and the protective role against atherosclerotic complications [189]. Presently, histological, fate mapping, and single-cell RNA sequencing studies suggest that smooth muscle cells play a major role in the pathogenesis of atherosclerotic plaques. Whether macrophage-like smooth muscle cells exert a protective or pro-atherosclerotic influence, and the regulatory mechanisms governing this phenomenon, are yet to be fully comprehended [190].

### Lipodystrophy syndromes

Lipodystrophy syndromes represent a diverse group of fat storage disorders, characterized by the inability to maintain subcutaneous body fat, leading to an aberrant and excessive distribution of body fat, accompanied by manifestations, such as diabetes, dyslipidemia, and CVD. The dysregulation of proteins or mutations in genes related to the formation or maintenance of lipid droplets are underlying mechanisms of lipodystrophy syndrome (reviewed in Refs. [191, 192]). Loss-of-function mutations in genes for triglyceride biosynthetic enzymes, encoded by *AGPAT2* (1-acylglycerol 3-phosphate o-acyltransferase 2) or *Lpin 1* (encoding lipin 1, phosphatidic acid phosphatase) result in lipodystrophy syndrome [192]. Lipin 1 expression is decreased in failing human hearts [193]. Cardiac-specific deletion of lipin1 in mice resulted in normal systolic function and mild cardiac hypertrophy with increased phosphatidic acid content as well as unexpected increases in diglycerides and triglycerides in the heart [193]. Frameshift mutations in the *PLIN1* gene, encoding perilipin 1 (a lipid droplet surface protein that regulates lipolysis), result in autosomal dominant partial lipodystrophy associated with severe dyslipidemia and diabetes [194]. In addition to enzymes that synthesize or regulate neutral lipids core, mutations in *CCTα* (CTP: phosphocholine cytidylyltransferase-α) that encodes an enzyme that regulates lipid droplets expansion by synthesizing phosphatidylcholine, cause lipodystrophy syndromes [195, 196]. Seipin is the ER membrane protein critical for initiating cytoplasmic lipid droplets formation and maintaining the contact site between ER and lipid droplets. Hereditary *seipin* deficiency causes the severe phenotype of lipodystrophy [197]. Furthermore, mutations in *FIT2* (Fat storage-inducing transmembrane protein 2, an ER membrane protein critical for lipid droplet biosynthesis) may be associated with lipodystrophy [198].

## Therapeutic targets and interventional strategies for lipotoxicity

Lifestyle modifications with diet and physical activity interventions lose weight, which improves lipid metabolism, inflammation, and cardiometabolic health. However, the efficacy of medical and surgical weight loss interventions shows mixed results for ischemic heart disease and heart failure. Lifestyle interventions are recommended for individuals of all ages, including children, adolescents, and adults, throughout life for not only primary prevention of CVD but overall healthy life, preceding pharmacotherapy [199]. Here we discuss pharmacotherapy aimed at targeting lipotoxicity.

### Drugs for diabetes and dyslipidemia

Metformin is the first-line medication for type 2 diabetes, followed by consideration of a sodium-glucose cotransporter 2 inhibitor (SGLT2i) or a glucagon-like peptide-1 receptor agonist. An increasing body of evidence indicates that metformin exerts its influence on multiple organs, including the liver, gut microbial communities, and tissue-resident immune cells, where mitochondria and lysosomes are the primary organelles targeted to achieve the glucose-lowering effect [200]. Metformin treatment reduces lipid accumulation in transplanted donor hearts of diabetic recipients when compared to those not receiving metformin [174]. A well-documented mechanism of metformin action involves the activation of AMP-activated protein kinase (AMPK), partly through transient inhibition of complex I, which stimulates fatty acid oxidation, thereby mitigating lipotoxicity in the liver, adipose tissue, and heart (reviewed in Ref. [201]). Metformin is accumulated in the gut, where it inhibits the intestinal absorption of dietary glucose. Importantly, metformin exerts an acute glucose-lowering effect in liver-specific [201] and intestine-specific [202] AMPK knockout mouse models, indicating the presence of AMPK-independent mechanisms (reviewed in Ref. [200]). These mechanisms involve fructose-1,6-bisphosphatase-1 (FBP1) [203], hepatic glucagon signaling [204], and changes in gut microbiota composition. However, metformin administration fails to ameliorate HFD-induced obesity and glucose intolerance in intestine-specific AMPKα1 knockout mice [205]. This paper demonstrated that metformin activates intestinal AMPKα1, which regulates brown adipose tissue thermogenesis by modulating gut microbiota composition and their metabolites, suggesting that AMPK activation in intestinal epithelial cells is required for the therapeutic effects of chronic metformin administration in mice fed a HFD. Recent independent two studies demonstrated that metformin exerts its effects in intestinal epithelial cells to increase the biosynthesis of the anorexigenic (appetite-suppressing) metabolite *N*-lactoyl-phenylalanine (Lac-Phe) through the inhibition of complex I, thereby leading to anti-obesity effects in cells *in vitro*, in mice *in vivo*, and in individuals regardless of the presence of diabetes [206, 207]. Lysosomes are another targeted organelle of metformin [208]. A recent study showed that metformin binds to presenilin enhancer 2 (PEN2) in the lysosomes of hepatocytes, which is recruited to ATPase H^+^ transporting accessory protein 1 (ATP6AP1), leading to the inhibition of v-ATPase and the activation of AMPK at the surface of lysosomes [209]. Whether metformin acts through the same mechanism involving the lysosomal pathway to target lipotoxicity in cardiomyocytes in the heart remains to be elucidated.

Recent clinical trials evaluating the impact of SGLT2i on CVD mark significant milestones in the treatment of heart failure. SGLT2i demonstrates a notable reduction in the risk of hospitalization for heart failure or cardiovascular death in patients with heart failure and a reduced ejection fraction (HFrEF) [210, 211] as well as in patients with heart failure with mildly reduced or preserved ejection fraction [212, 213], regardless of the presence or absence of diabetes. These findings suggest the presence of an anti-diabetes-independent cardioprotective mechanism associated with SGLT2i. Furthermore, a comprehensive meta-analysis of five randomized controlled trials (DELIVER, EMPEROR-Preserved, DAPA-HF, EMPEROR-Reduced, and SOLOIST-WHF) has confirmed that SGLT2i displays substantial benefits for cardiovascular death and hospitalization for heart failure irrespective of left ventricular ejection fraction [214]. However, the precise mechanisms governing the cardioprotective actions of SGLT2i in heart failure remain largely indeterminate. One of the proposed mechanisms suggests that SGLT2i induces a physiological level of ketosis [215]. Metabolomics analysis of myocardium samples obtained from end-stage human failing hearts [216], quantitative mitochondrial proteomics on myocardium samples from a hypertrophic heart failure mouse model [217], and metabolomics profiling of blood samples obtained from the artery and coronary sinus of patients with or without heart failure [218] collectively revealed increased utilization of ketone bodies and reduced utilization of fatty acids in the failing hearts. Furthermore, findings from a rodent experiment using a mouse model with a disrupted ketolysis indicate that the upregulation of ketone utilization serves as an adaptive mechanism in response to cardiac hypertrophy and heart failure [219]. Ketone bodies have been demonstrated to serve as a viable fuel source for failing heart in an isolated working mouse heart model [220, 221]. The infusion of exogenous 3-hydroxybutyrate (3-OHB) for 3 h demonstrated favorable hemodynamic effects, as assessed by Swan-Ganz catheterization and echocardiography, in patients with HFrEF [222]. A randomized, controlled, double-blind trial demonstrated that bolus administration of ketone ester improves cardiac output and left ventricular ejection fraction in patients with cardiogenic shock [223]. Furthermore, supplementing ketone bodies through a low-carbohydrate ketogenic diet exhibited improvements in cardiac hypertrophy and heart failure in mice subjected to acute pressure overload, in part through the suppression of the mTOR pathway by ketone-mediated activation of AMPK [224]. These findings suggest that SGLT2i exerts cardioprotective effects in both HFpEF and HFrEF, partially through the induction of a therapeutic range of ketosis. In addition to the modulation of ketone metabolism, a recent *in vitro* study employing metabolomics, lipidomics, and proteomics analyses revealed that empagliflozin, an SGLT2i, influences lipid accumulation, including the restoration of DHA levels, in the AC16 human cardiomyocyte cell line treated with high glucose [225], suggesting a cell-autonomous effect of SGLT2i on mitigating lipotoxicity.

Statins (HMG-CoA reductase inhibitors) are the first-line medication for primary and secondary prevention of cardiovascular death and hospitalization for heart failure in patients with ASCVD without heart failure [226]. The anti-inflammatory and lipid lowering effects of statins may also provide benefits for diabetic cardiomyopathy; however, randomized controlled clinical trials have yet to substantiate the cardiovascular benefits of statin usage in patients with heart failure [227, 228]. Conversely, it is noteworthy that a meta-analysis [229], a retrospective cohort study based on data from the Kaiser Permanente Southern California [230], and a recent retrospective database analysis of large single health care practice [231] have suggested a potential benefit of selectively using statins for clinical outcomes in patients with HFrEF and ASCVD or in patients with HFpEF [230] in real-world clinical settings. Future research is required to determine the benefits of statin usage in patients with heart failure, particularly those with non-atherosclerotic CVD origins, such as diabetic cardiomyopathy.

Recent four clinical trials focusing on triglyceride-lowering therapy have yielded mixed results for CVD outcomes. In the REDUCE-IT trial, patients with established CVD or diabetes who received statins and exhibited elevated triglyceride levels showed a favorable effect from daily consumption of 4 g of icosapent ethyl (an eicosapentaenoic acid ethyl ester) against cardiovascular events than the placebo group [232]. In contrast, in the VITAL trial, supplementation with marine n-3 (also known as omega-3) fatty acids at a dose of 840 mg per day did not significantly lower the incidence of cardiovascular events [233]. Furthermore, in the STRENGTH randomized clinical trial, Epanova, a carboxylic acid formulation of omega-3 fatty acids eicosapentaenoic acid (EPA) and docosahexaenoic acid (DHA), failed to reduce the incidence of cardiovascular events in statin-treated participants with established ASCVD or those with hypertriglyceridemia and low HDL cholesterol levels [234].

Fibrates, small molecule ligands of PPARα, have been clinically used to reduce circulating triglyceride levels. The extended follow-up study of the ACCORD Lipid Trial (ACCORDION) has demonstrated that fenofibrate effectively reduces cardiovascular events in diabetic patients treated with statins [235], indicating that fibrates may be beneficial for CVD independently of statin therapy. However, in a recent multinational, double-blind, randomized, controlled trial (PROMINENT trial), Pemafibrate, a selective PPARα modulator that has greater triglyceride-lowering and high-density lipoprotein (HDL) cholesterol-raising properties than other fibrates [236], failed to reduce cardiovascular events (a composite of nonfatal myocardial infarction, ischemic stroke, coronary revascularization, or cardiovascular death) in patients with hypertriglyceridemia and diabetes undergoing statin treatment (where 96% of patients were receiving statins), compared to the placebo group, despite successfully improving hypertriglyceridemia and low HDL cholesterol levels [237]. Pemafibrate, in addition, did not contribute to a reduction in hospitalizations for heart failure. These findings suggest that triglyceride-lowering therapy may not provide additional benefits for ASCVD events beyond statin treatments. Further studies are required to confirm whether triglyceride lowering by fibrates or omega-3 may play a role in mitigating the risk of non-atherosclerotic CVD, including diabetic cardiomyopathy.

### Targeting gut homeostasis

Obesity and obesity-related comorbidities correlate with richness of human gut microbiome [56]. Observational studies identified associative effects of drugs, drug combinations, and previous exposure to antibiotics on variations in the gut microbiome in patients with cardiometabolic disease [84]. One such drug is metformin. The *in vitro* gut-simulator experiments, coupled with transcriptome and gene ontology analyses, revealed that metformin directly modulates the composition of gut microbiota, partially by regulating the expression of genes encoding metalloprotein or metal transporters in individual bacterial species [238]. These findings suggest that metformin may not directly target cardiac steatosis but may achieve the effects through the modulation of gut microbiota populations. In addition to pharmaceutical interventions, the intake of dietary nitrate (NO_3_^−^) through beetroot juice has been shown to enhance exercise capacity in patients with HFpEF [239] and increase the maximum rate of oxygen consumption during exercise in patients with diabetes [240]. Rodent studies demonstrated the beneficial effect of dietary nitrate on HFD-induced liver steatosis through the modulation of microbiota [241] and cardiomyopathy [242] in mice. These findings suggest that dietary nitrate could be a therapeutic option for diabetic cardiomyopathy through the modulation of gut microbiota. Notably, the fecal metagenomic analysis of the cross-sectional MetaCardis Body Mass Index Spectrum cohort identified statin therapy as a key covariate of microbiome diversification and revealed a negative association between obesity-related microbiota dysbiosis and statin treatment [243]. Given that statin treatment may be ineffective for chronic heart failure in the absence of ASCVD, it is imperative to acknowledge that beyond the modulation of gut microbiota diversity through drugs or dietary interventions, certain specific bacterial species or their bioactive products may play a critical role in lipotoxicity-associated CVD. If this is the case, it is crucial to ascertain the identities of these entities.

### Targeting alternative splicing

Antisense oligonucleotides (ASOs), synthetic single-stranded short RNA or DNA molecules that control alternative splicing, have obtained clinical approval from both the United States Food and Drug Administration (FDA) and the European Medicines Agency (EMA) for application in hereditary diseases, including spinal muscular atrophy (SMA). Nusinersen, a modified ASO drug, modulates pre-mRNA splicing of the survival motor neuron 2 (SMN2) gene, thereby promoting the expression of full-length SMN protein. A multicenter, double-blind, sham-controlled trial showed that nusinersen significantly improves motor function compared with sham control group in infants with SMA [244] and children with later-onset SMA [245]. Clinical trials that assess the efficacy of ASOs for improving lipid metabolism have provided positive outcomes on lipid parameters. For example, Vupanorsen, an ASO drug that inhibits the synthesis of Angiopoietin-like 3 (ANGPTL3) protein, demonstrated a notable reduction in non-HDL cholesterol levels in patients with dyslipidemia under statin treatment [246]. Future pre-clinical and clinical studies would be needed to determine whether ASO treatment confers beneficial effects on diabetic cardiomyopathy associated with alternative splicing.

## Discussion

Although much progress has been made in elucidating the underlying mechanisms of diets or obesity-mediated lipotoxicity, many issues remain to be investigated. First, to date, the majority of studies on the biology of lipid droplets have been extensively conducted in adipose tissue and the liver. However, the roles and functional consequences of their dysregulation, including impaired lipid droplet formation and lipolysis, in cardiomyocytes remain largely unexplored. Considering the cell and tissue-specific regulation of lipid droplets, future research employing *in vitro* cardiomyocyte models is required. *In vitro* experiments using human cardiomyocytes derived from induced pluripotent stem cells may provide new insights into the mechanisms of lipotoxicity in a species- and cell type-specific manner. Additionally, it is crucial to ascertain the functional significance of the proposed mechanism in the development of diabetic cardiomyopathy *in vivo*.

Second, in certain cases, lipotoxicity tends to preferentially emerge in smooth muscle cells or macrophage, potentially manifesting as ASCVD (e.g., coronary artery disease). Conversely, in other cases, lipotoxicity arises in cardiomyocytes, giving rise to diabetic cardiomyopathy. Alternatively, it is conceivable that the progression of lipotoxicity may occur uniformly in both cell types, leading primarily to coronary artery disease rather than diabetic cardiomyopathy in humans. The susceptibility of each cell type to lipid overload, toxic lipid species, or inflammatory cytokines may be determined by RNA modifications, including alternative splicing, epigenetic modifications, and genetic factors. In this case, future research is warranted to understand the underlying mechanisms by which DNA, RNA, or histone modifications confer cell type-specific effects of lipotoxicity in the cardiovascular system. It may also be possible that a particular fatty acid or composition of fatty acids could induce lipotoxicity exclusively in cardiomyocytes. If so, there would be a necessity to identify a toxic lipid species that is cell type-specific and to elucidate the underlying molecular mechanism governing its release from lipid droplets or the gut microbiota, as well as how it damages cardiomyocytes.

Finally, while toxic lipid accumulation specifically in the heart is sufficient to induce diabetic cardiomyopathy, it remains uncertain whether exclusively targeting cardiac steatosis is sufficient for sustained improvement in diabetic cardiomyopathy over the long term. Given the complexity of systemic lipid metabolism involving factors, such as diets, physical activity, gut microbiota, medications, circulating fatty acids, cellular signaling, and DNA/RNA/histone modifications, it is conceivable that merely inhibiting cardiac lipotoxicity may not yield sustained long-term benefits for diabetic cardiomyopathy. In this regard, it is imperative to ascertain the systemic effectiveness of pharmacological approaches that target the core lipid droplet machinery or lipid-lowering drugs for obesity or diabetic cardiomyopathy in real-world clinical settings.

## Footnotes

1
https://www.who.int/news-room/fact-sheets/detail/obesity-and-overweight
